# Global Contraceptive Failure Rates: Who Is Most at Risk?

**DOI:** 10.1111/sifp.12085

**Published:** 2019-02-21

**Authors:** Sarah E.K. Bradley, Chelsea B. Polis, Akinrinola Bankole, Trevor Croft

## Abstract

Contraceptive failure is a major contributor to unintended pregnancy worldwide. DHS retrospective calendars, which are the most widely used data source for estimating contraceptive failure in low‐income countries, vary in quality across countries and surveys. We identified surveys with the most reliable calendar data and analyzed 105,322 episodes of contraceptive use from 15 DHSs conducted between 1992 and 2014. We estimate contraceptive method‐specific 12‐month failure rates. We also examined how failure rates vary by age, education, socioeconomic status, contraceptive intention, residence, and marital status using multilevel piecewise exponential hazard models. Our failure rate estimates are significantly lower than results from the United States and slightly higher than previous studies that included more DHS surveys, including some with lower‐quality data. We estimate age‐specific global contraceptive failure rates and find strong, consistent age patterns with the youngest users experiencing failure rates up to ten times higher than older women for certain methods. Failure also varies by socioeconomic status, with the poorest, and youngest, women at highest risk of experiencing unintended pregnancy due to failure.

## INTRODUCTION

Contraceptive failure is a major contributor to unintended pregnancy around the world, and represents a gap between women's and couples’ intentions to avoid pregnancy and their ability to implement those intentions. Elimination of that gap is a goal of policies and programs worldwide (Brown et al. 2014; Galati 2015). Despite the programmatic and demographic significance of contraceptive failure, remarkably little is known about its correlates, especially outside of high‐income countries. Recent studies in the United States and France have generally found contraceptive failure rates to decrease as strength of motivation to avoid pregnancy increases, and as socioeconomic status increases. Results are inconsistent, however, and vary by contraceptive method selected (Moreau et al. 2007; Kost et al. 2008; Black et al. 2010). In low‐ and middle‐income settings, two multicountry studies modeled correlates of contraceptive failure using Demographic and Health Survey (DHS) data from the 1980s and early 1990s (Moreno 1993; Curtis and Blanc 1997). More recently, two studies estimated contraceptive failure based on data from a number of more recent DHS surveys (Ali, Cleland, and Shah 2012; Polis et al. 2016).

We recently published a report estimating failure rates from the most recent DHS survey in 43 countries (Polis et al. 2016), acknowledging a prior analysis of DHS calendar data quality (Bradley, Winfrey, and Croft 2015). We concluded that some failure rates in our earlier (Polis et al. 2016) report were likely underestimated, due to underreporting of contraceptive episodes in the calendars of some surveys. We estimated contraceptive failure rates by binary groupings of sociodemographic characteristics (e.g., age <25 and 25+; parity 0–2 and 3+; primary education or below and secondary+ education), but we did not examine finer categorizations, nor correlates of failure in a multivariate framework (Polis et al. 2016).

In the present study, rather than analyzing the widest range and most recent data possible, we focus on a smaller number of surveys that we believe most accurately represent women's reproductive experiences, trading comprehensiveness (and in some cases, survey recency) for data reliability concerns noted in Polis and colleagues (2016). We evaluate the reliability of calendar data in every DHS survey that collected the necessary calendar data and was made publicly available on the DHS program website as of January 2016. We pool together the 15 surveys judged to have the most reliable data, drawn from a wide range of low‐ and middle‐income countries. We also test whether limiting our analysis to the most reliable surveys conducted in the past 10 years, or limiting the calendar recall period (described below) to a single year, changes estimates of contraceptive failure.

By pooling episodes of contraceptive use for the same method across multiple surveys with reliable data, we are able to produce finely disaggregated estimates of failure, including age‐specific failure rates for implants, IUDs, injectables, pills, condoms, withdrawal, and periodic abstinence, as well as method‐specific multilevel hazard models to examine correlates of contraceptive failure in a multivariate framework.

### Age and Contraceptive Failure

Age‐specific failure rates by contraceptive method can provide an important contribution to our understanding of contraceptive use dynamics. A priori, we would expect to see large variations in failure rates by age for multiple reasons. One, women's biologic fecundity, or the probability of conception per coital act, decreases with increasing age (Menken, Trussell, and Larsen 1986), as does their male partner's (Kuhnert 2004; Matorras et al. 2011). Two, coital frequency also decreases with age (Westoff 1974). Three, older contraceptive users are likely to have more experience using the method and may be less likely to experience failures due to method unfamiliarity. However, patterns of failure by age have not always followed these expectations for all methods. In France, the hazard of condom failure was higher among women aged 20–34 than among teenagers (Moreau et al. 2007). In the United States, contraceptive failure rates for all methods combined were lower for women 30+ compared to women in their twenties (Kost et al. 2008). A subsequent US analysis assessing specific methods reported similar age patterns for IUDs, pills, and other hormonal methods, but reported no differences in failure rates by age for condoms, withdrawal, and all reversible methods combined (Sundaram et al. 2017). In Polis and colleagues (2016), women under 25 had significantly higher failure rates than women aged 25+ for every method except implants, which had a failure rate of 0.6 pregnancies per 100 use episodes in both age groups. We found the lack of consistent age patterns in previous analyses of failure rates surprising, and investigate these patterns in more detail with this rich dataset.

### Other Correlates of Contraceptive Failure

We reviewed known factors associated with contraceptive failure, as described in an analysis of DHS data from 43 countries globally (Polis et al. 2016), an analysis using the most recent nationally representative data from the United States (Sundaram et al. 2017), and a review of literature on factors associated with contraceptive failure (Black et al. 2010). All sources found some correlations between age and contraceptive failure, with the exceptions noted above. Union status may also be associated with contraceptive failure, with higher failure rates observed among never‐married women (versus ever‐married women) for most methods except condoms (for which the opposite pattern occurred) internationally (Polis et al. 2016), and higher failure for cohabitating or formerly married women versus married women across all methods combined, in the United States (Sundaram et al. 2017).

The association of contraceptive failure with parity varied internationally and in the United States, with higher failure for some methods among lower‐parity women internationally, but higher failure for some methods (pills, condoms, withdrawal, and all hormonal methods combined plus IUDs) for higher‐parity women in the United States (Sundaram et al. 2017). Internationally, women using contraception to space (versus to limit) births tended to have higher failure rates, though estimates did not vary significantly by intention for implants, IUDs, or oral contraceptives. These patterns held regardless of parity for user‐dependent methods, but higher‐parity IUD and pill users who were limiting had higher contraceptive failure than higher‐parity IUD and pill users who were spacing (Polis et al. 2016). Women with less motivation to avoid pregnancy may both be more likely to use a method inconsistently and more likely to use less reliable methods (Black et al. 2010).

The association of wealth with contraceptive failure was similar in various geographical contexts, with higher failure rates occurring among poorer women, except for user‐independent methods such as implants, IUDs, and injectables—and in the international setting, this association held regardless of age (Black et al. 2010; Polis et al. 2016; Sundaram et al. 2017). In the international analysis, contraceptive failure was not associated with urban versus rural residence, except that urban injectable users had higher failure rates than rural injectable users. Similarly, education did not appear to be strongly associated with contraceptive failure for most methods (Polis et al. 2016).

In the United States, black women and Hispanic women had higher failure rates than white women or women of other races for some user‐dependent methods (Sundaram et al. 2017). A number of other contextual factors that have not often been specifically examined in analyses of contraceptive failure may play a role, including higher coital frequency, substance abuse, interactions between medications and hormonal contraceptive methods that may impact effectiveness or cause unexpected side effects, relationship violence, incorrect information or misperceptions about correct use stemming from miscommunication between providers and patients, barriers to access to contraceptive services, and impacts from side effects or a higher body‐mass index (Black et al. 2010).

## DATA AND METHODS

We use data from 15 Demographic and Health Surveys, which are large‐scale, nationally representative household surveys of women of reproductive age (15–49). In the surveys selected, participants were asked about pregnancies, births, terminations, and episodes of contraceptive use that occurred over the past five or more years, producing a retrospective month‐by‐month reproductive calendar history for each woman, hereinafter referred to as “calendar data.” For each episode of contraceptive use that was discontinued in the calendar period, women were asked, “Why did you stop using the (method)?” Women's responses are categorized into one of 14 precoded categories, including “became pregnant while using” (i.e., reported contraceptive failure). These histories allow for the use of life table methods to calculate failure rates by contraceptive method. The failure rates in this article represent typical‐use, rather than perfect‐use, failure rates, including both method‐related failures (failure of the method to work as expected) and user‐related failures (stemming from incorrect and/or inconsistent use of the method).

### Selection of Datasets Included in Analysis

The collection of retrospective calendar data requires women to accurately recall episodes of contraceptive use that occurred up to seven years in the past. Women may omit failures that occurred long ago simply due to recall biases; they may report they ended contraceptive use for reasons other than failure due to social desirability bias; or they may omit episodes of use that ended in a failure to avoid discussing the failure, especially if the failure ended in an abortion. Accurate recall may be particularly difficult for contraceptive methods that are used only sporadically, such as coitus‐dependent methods. Recall may also be more difficult for older women, who have generally been sexually active for a longer time and thus need to recall episodes of use further back in time, compared to adolescents who may have only become sexually active recently. Underreporting of retrospective contraceptive use in the calendar occurs in an estimated 74 percent of comparisons between calendar data and current‐use estimates for the same time point (Bradley et al. 2015). To obtain the most reliable estimates from imperfect data, we used multiple strategies to identify the surveys likely to be of highest quality and to limit the impact of potential biases.

First, we selected surveys in which the calendar data could be validated against external information (comparisons with current‐status method‐specific contraceptive prevalence rate (CPR) data from previous DHSs, as described in Bradley and colleagues 2015). We only included surveys that showed no evidence of underreporting of any of the contraceptive methods analyzed here. This stringent selection criterion excluded more than 60 percent of surveys considered for potential inclusion. The lack of evidence of underreporting in the surveys we selected indicates that few, if any, episodes of contraceptive use were omitted due to recall, social desirability, or other biases.

Second, we examined each survey for other types of misreporting, calculating indices for multiple data quality measures including potential underreporting, heaping, and displacement of events in the reproductive calendars for each contraceptive method analyzed in each survey, described in detail in Addendum A. We examined the distribution of each index for outlying values. We considered any value greater than p75 + 1.5^*^interquartile range as an outlier. We excluded surveys that had outlying values in the upper tails of any of these indices. This exclusion leaves us with surveys in which women are apparently able to correctly remember contraceptive use episodes and place them accurately in time, rather than heaping their start dates on convenient months such as January, for example. If there were multiple surveys within a country that fit these selection criteria, we selected the most recent survey. These selection criteria led to a sample of 15 surveys: Armenia 2005, Bangladesh 2011, Colombia 2010, Dominican Republic 1996, Egypt 2014, Honduras 2011–12, Jordan 2009, Kenya 1998, Morocco 1992, Peru 2012, Philippines 2003, Rwanda 2010, Senegal 2012–13, Turkey 2003, and Zimbabwe 2005–06. Use of different selection criteria would clearly produce a different survey sample. For this analysis, however, we felt comfortable using this most restrictive set of selection criteria, which we believe indicates the highest‐quality survey data. The selected surveys come from a wide range of socioeconomic and demographic contexts in Africa, Asia, Eastern Europe, and Latin America.

Third, we considered which portions of the retrospective calendar—spanning a period of between five and seven years before the date of interview—would be most reliable. According to an earlier study, contraceptive use was most poorly reported for points furthest back in time (Bradley et al. 2015) suggesting that resulting failure rates using information from such periods were most likely to be underreported also. Under the theory that contraceptive failure rates for an individual method should not change dramatically within the same country across a single five‐year period, we tested this concept by splitting each calendar period (typically 5 to 7 years; see Bradley et al. 2015 for details) into two equal time segments and calculating single‐decrement failure rates separately for each time segment. In the majority of comparisons, contraceptive failure rates were substantially lower when estimated from the early time segment versus the later time segment within each survey. Although this pattern was not found in every survey, it does suggest that contraceptive failures are frequently underreported for periods further back in time. The finding further suggests that the problems with underreporting of contraceptive use episodes do affect estimates of contraceptive failure and, most likely, discontinuation rates for other reasons. We therefore decided to use only the most recent data from each survey. We exclude the most recent three months from analysis because women in their first trimesters may not yet recognize they are pregnant, which could lead to underestimation of failure rates. We use the 3–38‐month period prior to each woman's interview as the window of observation for analysis.

The final sample using the most recent 3–38 month calendar segments from 15 surveys yielded 105,322 episodes of contraceptive use collected from 97,094 women interviewed.

We conducted sensitivity analyses to test whether the inclusion of older surveys—those conducted more than 10 years ago—had any effect on the results by recalculating estimates, limiting the data to surveys conducted since 2008. We also tested whether using the 3‐year recall period versus a shorter 1‐year recall period changed the results of our analyses. Full details are shown in Appendix Tables 1–4^1^ and described in the Sensitivity Analyses section.

### Analytic Methods

Each segment of contraceptive use reported in the reproductive calendar was converted to a contraceptive episode for analysis. A single woman could contribute multiple episodes to the analysis if she stopped and started using contraception several times during the calendar period, or switched between different methods. Each episode is a segment of a single method of use. If multiple methods are used at the same time, the most effective method is recorded in the survey (ICF 2018). To calculate failure rates, we constructed episode‐based associated single decrement life tables (Preston, Heuveline, and Guillot 2000). In these calculations, all contraceptive discontinuations for reasons other than contraceptive failure were censored. Episodes of use that began prior to this window enter into the life table as late entries (see Polis et al. 2016 for details of life table calculations and left truncation). Details of these calculations and use of the Delta Method for confidence interval calculations are included in Addendum B.

To analyze factors associated with contraceptive failures, we used multilevel piecewise exponential hazard models. We partitioned the duration of contraceptive use into intervals *s* within which the baseline hazard is assumed to be constant. Based on graphical analyses of the baseline hazards and following previous analyses of contraceptive failure (Curtis and Blanc 1997; Moreau et al. 2007; Bradley, Schwandt, and Khan 2009), we defined intervals of three months duration for the first year of use (0–2, where month 0 is the month of uptake; 3–5; 6–8; and 9–11 months).

In preliminary analyses, we found that the baseline hazard was far more similar for the same method across countries than for different contraceptive methods within the same country. We therefore pooled all data together across countries, and estimated separate models (both unadjusted failure rate models and multilevel hazard models) for each of the seven most commonly used contraceptive methods: pills (combined or progestin‐only), injectables (combined or progestin‐only), IUDs (hormonal and nonhormonal), implants,^2^ male condoms, withdrawal, and periodic abstinence.^3^ Using separate models for each contraceptive method is in line with previous findings that different types of women select into using different contraceptive methods (WHO Task Force 1980; Frost and Darroch 2008). Frost and Darroch (2008) found that socioeconomic, demographic, and partner characteristics were significant predictors of the methods women chose to use, and that women with strong motivation to avoid pregnancy were more likely to choose more effective reversible methods. The WHO Task Force (1980) found that urban/rural residence was strongly associated with method selection in India and Turkey, and that women who intended to space, rather than limit, were more likely to select less effective short‐acting methods, rather than IUDs in India, Korea, and Turkey. Further, Steele and Curtis (2003) found that method choice is endogenous with some types of contraceptive discontinuation.

Data from all interviewed women in each of the selected surveys were pooled together for analyses described below. All analyses are weighted using sampling weights, and weights were multiplied by a survey‐specific constant defined as $\frac{\sum_{1}^{n}w_{i}\;}{n\;w_{i}}$, where *w_i_* is the weighted number of interviewed women in survey *i*, and *n* = 15 surveys included in analysis. This constant equalizes the effective weighted sample size across surveys, so each survey contributes equally to the analysis, i.e., results are not weighted more heavily toward surveys with larger sample sizes. Results are therefore interpretable as averages across all women in the surveys included in analysis.

Episodes of contraceptive use were linked with data from other sections of each woman's individual interview, allowing for examination of failures by demographic and socioeconomic characteristics. We measure age at the start of the episode of use, grouping women's ages into 5‐year categories with an open‐ended category for women aged 40 and older because failures are very rare among women in this age group.

Marital status during each episode is measured by comparing the date of the end of the contraceptive use episode to the date of the woman's (first) marriage. Each episode is then classified according to whether the woman had been married at the time of discontinuation, or whether she had never been married before she discontinued. For women who were married only once and report they are currently married at the time of survey, “ever married” is synonymous with currently married at the time of discontinuation. For formerly married women, however, we do not know the date of marital dissolution, and for women who were married more than once, we do not know the date of any marriage after the first. We therefore can only classify women as “ever married” or “never married” at the time of the episode of contraceptive use. If failure rates are substantially different between currently and formerly married women, this may lead to over‐ or under‐estimation of the failure rate for currently married women. We anticipate that women using to space, rather than limit (hereinafter called *contraceptive intention*) their childbearing, may experience higher levels of failure because the anticipated costs of a mistimed pregnancy are lower than an unwanted pregnancy.

Following Lightbourne (1985), contraceptive intention is calculated by comparing women's reported ideal number of children to the number of children they had when the episode of contraceptive use began. If their ideal number was less than or equal to their current number of children, women were assumed to have already achieved their ideal family size and the episode was classified as “using to limit.” All other episodes of use were classified as “using to space.” This includes non‐numeric responses to the question on ideal number of children, such as “up to God.” We reason that women who do not give a numeric ideal family size, but still use contraception, are using in order to space, rather than limit, their births. This classification allows contraceptive intention to be time‐varying with each episode of contraceptive use, but assumes that reported ideal number of children is constant over time, which may not be valid.

We include educational level, using DHS standard classifications of no education, primary, and secondary or higher education based on each country's educational system (MEASURE DHS 2013), and socioeconomic status as proxies of access to information and contraceptive services and supplies, anticipating that failure rates may be lowest among the wealthiest and most highly educated. For our socioeconomic status measure, we use the DHS “wealth index” constructed from information on household ownership of durable goods and amenities using principal components analysis, placing households on a continuous scale of wealth within the country, which is then divided into equally sized quintiles by population size (Rutstein and Johnson 2004).

These final two measures are country‐specific, as educational systems vary by country and wealth quintiles are relative only to other households within the same country. These two measures are only measured at the time of the survey, and are not time‐varying. We do not expect a great deal of mis‐specification associated with this limitation, however, as it is unlikely that large proportions of women will have experienced substantial changes in educational or socioeconomic status within the three‐year period prior to interview.

We fit multilevel models of contraceptive failure for each method using Poisson regression with the logarithm of the time each woman is at risk of failure within the 3‐month interval *s* as an offset (Rabe‐Hesketh and Skrondal 2012). The model is

$$\begin{array}{ccc} \log(\mu_{seij}) & = & \log\left(t_{seij}\right)+\alpha_{1s}+\alpha_{2j}+\beta_{1}X_{1seij}+\beta_{2}X_{2seij}\\ & & +\;\delta_{1}Y_{1eij}+\gamma_{1}{Z_{1}}_{ij}+\gamma_{2}{Z_{2}}_{ij}+\zeta_{ij} \end{array}$$

where $\mu_{seij}$ is the mean parameter of the Poisson distribution, $t_{seij}$ is the time at risk of failure in the 3‐month interval *s* for contraceptive episode *e* from the reproductive calendar of woman *i* in country *j*, $\alpha_{1s}$ is an interval‐specific intercept that allows the baseline hazard of failure for that contraceptive method to change every 3 months, $\alpha_{2j}$ is a country‐specific fixed effect, $X_{1seij}$ represents each woman's age at the beginning of interval *s*, $\;X_{2seij}$ represents her marital status at the beginning of interval *s*, $Y_{1eij}$ represents whether the intention of contraceptive use segment *e* was to space or limit births, ${Z_{1}}_{ij}$ measures the country‐specific highest educational level achieved by woman *i* at the time of the survey, ${Z_{2}}_{ij}$ is a measure of the woman's country‐specific socioeconomic status at the time of the survey, the random intercept $\zeta_{ij}$ introduces dependence among the hazards for different episodes of contraceptive use for the same woman *i*, and exp $\left(\zeta_{ij}\right)$ is assumed to be normally distributed and independent of the covariates. $\zeta_{ij}$ represents an unobserved frailty shared across contraceptive episodes for the same woman, measuring constructs such as women's underlying fecundity or propensity toward failure that is not captured by her age or other sociodemographic characteristics included in the model.

#### Sensitivity Analysis Methods

Although the DHS program has many processes in place to aid women's recall of contraceptive events—notably, asking first about the most significant reproductive events, including births, pregnancies, and terminations, and then asking about contraceptive use episodes as they relate to the time between, say, the birth of their first child and their subsequent pregnancy (ICF 2018)—there are still understandable concerns that women do not accurately recall episodes of contraceptive use that occurred long ago. We therefore conducted a sensitivity analysis to see if the analyses above changed when we limited the recall period to a single year—specifically, months 3–14 prior to interview, excluding the most recent 3 months to avoid underestimating failures due to unrecognized pregnancies in the first trimester. Results from these sensitivity analyses are shown in Appendix Tables 1 and 2.

We also note that some of the surveys included in analysis are more than 20 years old and may not accurately depict women's current experiences. This is particularly true in the Dominican Republic, Kenya, and Morocco—countries in which the most reliable data were found in surveys conducted in the 1990s. However, two studies that examined trends in failure rates using multiple DHS surveys from the same country found failure rates for each method to remain relatively consistent within each country over time (Bradley, Schwandt, and Khan 2009; Ali, Cleland, and Shah 2012). We tested whether limiting the analysis to surveys conducted in the last 10 years (since 2008) had an impact on the results. Results from these sensitivity analyses are shown in Appendix Tables 3 and 4.

In each sensitivity analysis, we checked whether each failure rate differed significantly from the rates in the main analysis, shown in Table 1, by checking whether the confidence intervals overlapped. Results that are significantly different from the main analysis are shown with an asterisk in the Significance column of Appendix Tables 1 and 3.

**Table 1:** Twelve‐month contraceptive failure rates by contraceptive method and sociodemographic characteristics, pooled data from 15 DHS surveys

	Implant			IUD			Injectable			Pill			Condom			Withdrawal			Periodic abstinence		
	Failure rate	(95% CI)	Number of episodes	Failure rate	(95% CI)	Number of episodes	Failure rate	(95% CI)	Number of episodes	Failure rate	(95% CI)	Number of episodes	Failure rate	(95% CI)	Number of episodes	Failure rate	(95% CI)	Number of episodes	Failure rate	(95% CI)	Number of episodes
Age groups																					
15–19	0.3	(0–1.3)	349	2.6	(1.6–4.3)	772	2.8	(2.2–3.6)	4,737	7.9	(6.8–9.1)	5,731	12.9	(10.8–15.5)	5,558	25.1	(21.8–28.7)	2,316	23.2	(19.6–27.4)	1,246
20–24	—	—	630	1.8	(1.1–3)	2,365	2.1	(1.7–2.7)	7,138	6.8	(5.9–7.7)	9,209	10.4	(8.2–13.1)	4,495	21.9	(19.1–25.1)	3,347	23.0	(19.8–26.8)	1,635
25–29	0.9	(0.2–3.3)	564	0.9	(0.6– 1.3)	2,549	1.7	(1.3–2.2)	5,802	5.9	(5.1–6.9)	7,839	8.0	(6.3–10.2)	3,161	17.9	(15.5–20.7)	3,185	23.6	(20–27.6)	1,691
30–34	0.3	(0.1–0.9)	375	1.1	(0.7–2)	1,748	2.3	(1.5–3.3)	3,620	6.0	(5–7.1)	4,847	6.2	(4.2–9)	2,066	12.9	(10.8–15.3)	2,135	17.5	(13.9–22)	1,467
35–39	—	—	201	0.5	(0.2–1)	945	1.5	(0.9–2.5)	2,050	5.8	(4.5–7.4)	2,567	4.4	(2.6–7.5)	1,281	11.6	(9–15.1)	1,310	13.0	(10.3–16.2)	1,068
40+	—	—	100	0.0	(0–0.6)	400	0.8	(0.2–2.7)	1,107	2.0	(1.2–3.2)	1,284	1.2	(0.6–2.4)	815	4.1	(2.2–7.5)	865	6.1	(3.9–9.5)	752
Highest education level																					
No education	0.7	(0.3–1.6)	214	0.3	(0.1–1.3)	659	2.1	(1.3–3.4)	2,129	6.9	(5.8–8.1)	3,380	6.1	(3.3–11.1)	325	13.2	(10.2–17.1)	772	17.1	(13.2–22)	505
Primary education	0.1	(0–0.4)	696	1.6	(0.9–3)	1,762	1.4	(1.1–1.7)	9,277	6.0	(5.2–7)	9,255	7.5	(5.8–9.6)	3,283	15.3	(13.7–17.1)	4,346	17.6	(15.2–20.3)	2,271
Secondary+ education	0.4	(0.1–1.6)	1,309	1.2	(0.9–1.5)	6,358	2.6	(2.2–3.1)	13,048	6.3	(5.7–7)	18,842	8.9	(7.8–10.1)	13,768	18.8	(16.9–21)	8,040	19.9	(17.8–22.1)	5,083
Socioeconomic status																					
Poorest	0.2	(0–1.2)	303	0.9	(0.5–1.8)	1,453	1.7	(1.3–2.2)	6,116	8.4	(7.1–9.9)	5,966	10.1	(7.8–13.2)	2,324	17.1	(14.7–19.9)	3,005	20.1	(16.9–23.8)	1,775
Second	—	—	443	1.8	(0.9–3.3)	1,733	2.3	(1.8–3)	6,154	7.0	(6.1–8.1)	6,864	10.2	(7.8–13.3)	3,462	19.4	(16.5–22.7)	2,916	23.0	(19.1–27.5)	1,667
Middle	0.5	(0.1–3.1)	497	0.9	(0.6–1.4)	1,833	2.1	(1.6–2.7)	5,167	6.6	(5.6–7.8)	6,627	11.5	(8.9–14.7)	3,809	17.1	(14.8–19.6)	2,736	16.9	(14–20.4)	1,538
Fourth	0.3	(0.1–1.8)	496	1.2	(0.8–2)	1,797	1.8	(1.2–2.7)	4,100	5.1	(4.3–5.9)	6,281	8.0	(6.5–10)	3,832	17.5	(14.5–21)	2,483	18.7	(15.5–22.3)	1,398
Highest	0.4	(0.2–0.9)	480	1.1	(0.7–1.9)	1,963	2.0	(1.3–3.1)	2,917	5.0	(4.1–6.1)	5,739	6.2	(4.8–7.9)	3,949	15.1	(12.5–18.2)	2,018	17.3	(14.3–21)	1,481
Contraceptive intention																					
Spacing	0.3	(0–1.3)	1,194	1.6	(1.2–2.2)	4,805	1.9	(1.6–2.3)	14,315	6.7	(6.1–7.3)	19,789	9.4	(8.3–10.8)	12,962	19.5	(17.8–21.4)	8,947	22.4	(20.3–24.6)	4,801
Limiting	0.4	(0.1–1.4)	1,025	0.7	(0.4–1)	3,974	2.1	(1.6–2.6)	10,139	5.7	(5.1–6.5)	11,688	6.4	(5–8.2)	4,414	13.0	(11.4–14.8)	4,211	13.8	(11.6–16.4)	3,058
Marital status																					
Never married	0.1	(0–2.1)	317	3.1	(1.4–6.5)	215	2.9	(2.1–3.9)	3,123	4.7	(3.5–6.2)	2,638	7.2	(6–8.6)	6,711	27.1	(21.4–33.9)	1,775	18.3	(15.1–22.1)	1,297
Ever married	0.3	(0.1–1)	1,902	1.2	(0.9–1.5)	8,564	1.9	(1.7–2.2)	21,331	6.4	(5.9–6.9)	28,839	8.9	(7.8–10.2)	10,665	16.7	(15.4–18)	11,383	19.1	(17.4–20.9)	6,562
**Total**	**0.3**	**(0.1**–**0.9)**	**2,219**	**1.2**	**(0.9**–**1.5)**	**8,779**	**2.0**	**(1.7**–**2.3)**	**24,454**	**6.3**	**(5.9**–**6.8)**	**31,477**	**8.6**	**(7.6**–**9.6)**	**17,376**	**17.3**	**(15.9**–**18.7)**	**13,158**	**19.0**	**(17.4**–**20.6)**	**7,859**

NOTE: Data come from contraceptive use episodes from the Armenia 2005, Bangladesh 2011, Colombia 2010, Dominican Republic 1996, Egypt 2014, Honduras 2011–12, Jordan 2009, Kenya 1998, Morocco 1992, Peru 2012, Philippines 2003, Rwanda 2010, Senegal 2012–13, Turkey 2003, and Zimbabwe 2005–06 DHS surveys, with the exposure period limited to months 3–38 prior to the survey.

— = Too few failures in that category to calculate estimates.

## RESULTS

### Failure Rates

**Figure 1:**
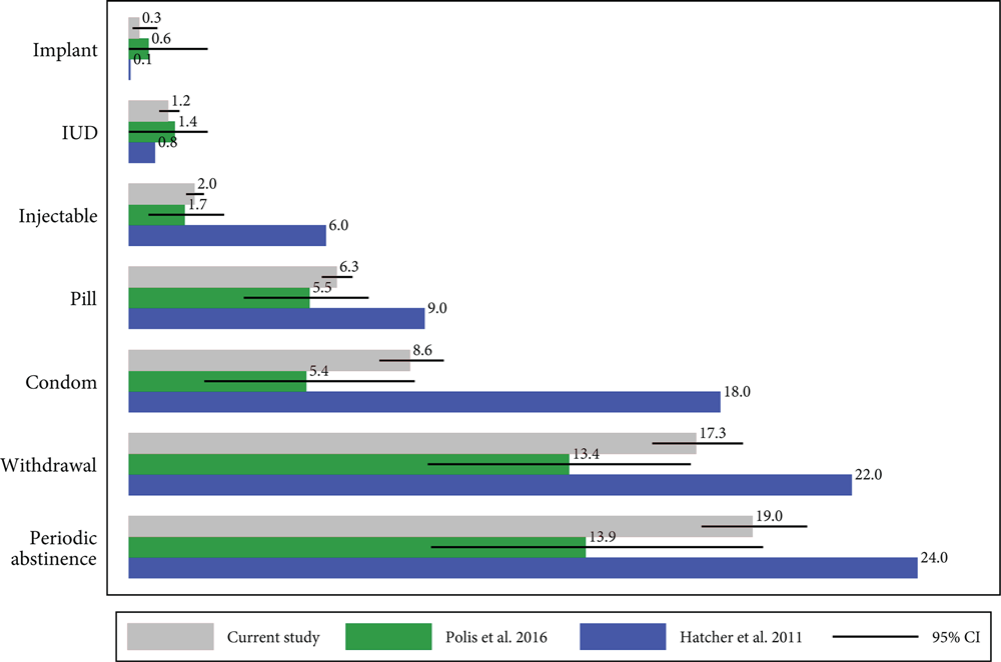
Twelve‐month failure rate estimates by contraceptive method from multiple studies

We display unadjusted 12‐month failure rates for each method in Figure 1 and Table 1. Twelve‐month failure rates are interpretable as the percentage of women who, on average,^4^ will become pregnant within the first year of typical method use. Estimates from the pooled sample used in this analysis, shown in the top bars in Figure 1, are extremely low for implants and IUDs, with approximately one woman out of 100 becoming pregnant across a one‐year horizon (implant failure rate = 0.3 per 100 episodes, 95% CI 0.1–0.9; IUD failure rate 1.2, CI 0.9–1.5). Approximately 2 percent of injectable users would be expected to become pregnant during the first year of use (95% CI 1.7–2.3). Failure rates are higher for short‐term resupply methods of pills and condoms, which require users to have the methods on hand and use them correctly, with an estimated 6 to 9 users out of every 100 becoming pregnant in the first year of use (pill failure rate 6.3, CI 5.9–6.8; condom failure rate 8.6, CI 7.6–9.6). The highest failure rates are seen for traditional methods of withdrawal and periodic abstinence, with 17–19 percent of users becoming pregnant within a year of beginning the method (withdrawal failure rate = 17.3, CI 15.9–18.7; periodic abstinence failure rate 19.0, CI 17.4–20.6).

Figure 1 compares the estimated failure rates from this study (in the top bars) with those we previously estimated from the median values of failure rates across 43 recent DHS surveys (Polis et al. 2016, in middle bars), and the widely cited typical use estimates from *Contraceptive Technology* based on US clinical and survey data from 1979, 1995, and 2002 (Hatcher 2011). In Polis and colleagues (2016), we noted that our estimated failure rates were similar to previous studies based on a broad range of DHS data (e.g., Ali, Cleland, and Shah 2012) but diverged markedly from estimates using US data. Specifically, these estimated failure rates were substantially lower than US estimates for injectables (1.7 versus 6), oral contraceptives (5.5 versus 9), male condoms (5.4 versus 18), withdrawal (13.4 versus 22), and periodic abstinence (13.9 versus 24). We noted that one potential source of this discrepancy is that the US estimates were corrected for abortion underreporting using secondary estimates of the number of abortions resulting from each contraceptive method from abortion patients surveys (Kost et al. 2008). No such information is available in the countries where DHS surveys are conducted, so DHS‐based results cannot be corrected in this way, however such adjustments would tend to increase, rather than decrease, estimated failure rates. The estimates both in the present study and in Polis and colleagues (2016) remain substantially lower than the Hatcher US estimates. For example, in the absence of the abortion underreporting correction, the 2002 US estimate of condom failure rate decreases from 17.4 to 13.9—still significantly higher than our present estimate of 8.6 (CI 7.6–9.6).

As shown in Figure 1, contraceptive failure rates estimated from calendar data that showed the lowest levels of underreporting (as described in Addendum A) are similar to the median rates across all available survey data for methods with low failure rates and thus limited variability, like implants and IUDs. Estimates of failure rates for implants and IUDs are virtually identical between the two data sources, with completely overlapping confidence intervals indicating that the results are not statistically significantly different. Our estimates of injectable failure are also similar (2.0 failures per 100 episodes of use versus 1.7), and reasonably similar for oral contraceptives (6.3 failures per 100 episodes of use versus 5.5). Differences between the present estimates and the estimates in Polis 2016 are larger for methods with higher levels of failure: condoms (8.6 versus 5.4), withdrawal (17.3 versus 13.4), and periodic abstinence (19.0 versus 13.9), though the current estimates fall within the upper bounds of the 95% confidence intervals of the 2016 analysis for condoms and periodic abstinence, and just beyond the upper bound of the 95% CI for withdrawal.

**Figure 2:**
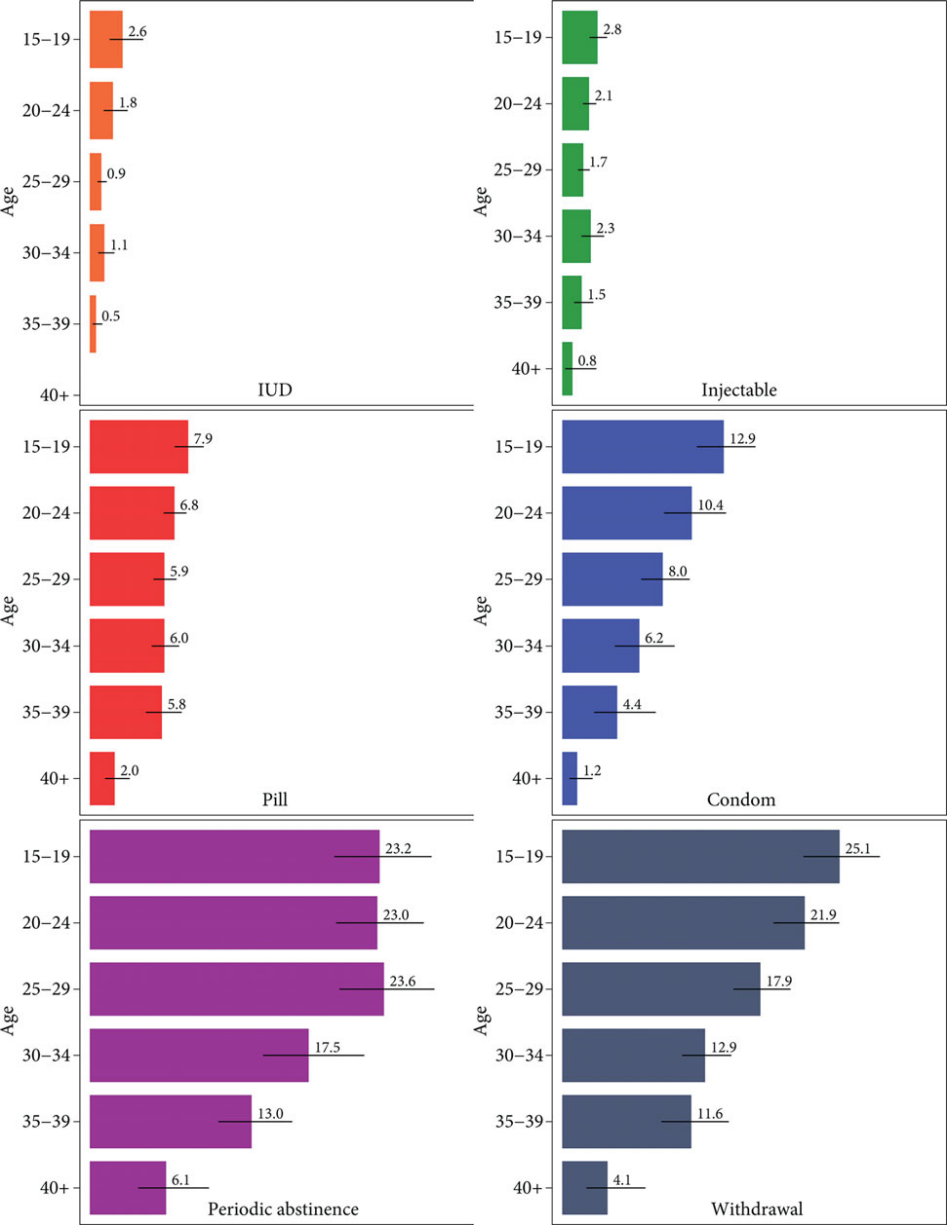
Twelve‐month failure rates by age and contraceptive method

As shown in Table 1, the strongest patterns in contraceptive failure are seen by age, with adolescents consistently experiencing the highest failure rates and women aged 40+ the lowest. Differences in failure estimates by age are substantial: condom users aged 15–19 experience contraceptive failure at more than 10 times the rate of women aged 40 and older (Figure 2). Pill and periodic abstinence users aged 15–19 have failure rates that are almost four times higher, and withdrawal users have failure rates that are six times higher, compared to women in their forties.

Failure rates for pills, condoms, withdrawal, and periodic abstinence are substantially higher for women in the poorest quintile of the population than for women from the wealthiest households, though results are only statistically significant for contraceptive pills (Table 1).^5^ The poorest pill users experience failure 8.4 times per 100 episodes of use (CI 7.1–9.9), while the wealthiest pill users have a significantly lower failure rate of 5.0 (CI 4.1–6.1). Women using contraceptives to space have significantly higher rates of contraceptive failure than those using to limit for IUDs (failure rate for spacing of 1.6, CI 1.2–2.2 versus limiting 0.7, CI 0.4–1.0), condoms (spacing 9.4, CI 8.3–10.8 versus limiting 6.4, CI 5.0–8.2), withdrawal (19.5, CI 17.8–21.4 versus 13.0, CI 11.4–14.8), and periodic abstinence (22.4, CI 20.3–24.6 versus 13.8, CI 11.6–16.4). Patterns of failure by education and marital status are inconsistent.

### Model Results

Table 2 presents results from the multilevel multivariate hazard models of contraceptive failure. In these models, age remains by far the most consistent predictor of contraceptive failure after adjusting for the other covariates. Compared to adolescent women aged 15–19, the hazard of failure for women aged 40 and older is 99 percent lower for IUD users, 64 percent lower for injectable users, 76 percent lower for pill users, 99 percent lower for condom users, and 76–81 percent lower for traditional method users. For most methods, the hazard of failure decreases monotonically as women's age increases.

**Table 2:** Adjusted hazard ratios of contraceptive failure within the first year of use by contraceptive method, pooled data from 15 DHS surveys

	IUD		Injectable		Pill		Condom		Withdrawal		Periodic Abstinence	
	Hazard ratio	95% CI	Hazard ratio	95% CI	Hazard ratio	95% CI	Hazard ratio	95% CI	Hazard ratio	95% CI	Hazard ratio	95% CI
Age <20 (r)	1.00		1.00		1.00		1.00		1.00		1.00	
20–24	0.95	(0.46–1.93)	0.93	(0.66–1.32)	0.83*	(0.68–1.02)	0.56***	(0.41–0.75)	0.86	(0.68–1.08)	0.82	(0.63–1.07)
25–29	0.61	(0.30–1.24)	0.69*	(0.45–1.07)	0.70***	(0.56–0.89)	0.35***	(0.25–0.51)	0.73**	(0.57–0.93)	0.85	(0.64–1.12)
30–34	0.85	(0.38–1.92)	1.05	(0.66–1.68)	0.69***	(0.52–0.91)	0.28***	(0.18–0.43)	0.54***	(0.41–0.72)	0.66**	(0.48–0.91)
35–39	0.40*	(0.15–1.05)	0.71	(0.39–1.28)	0.65**	(0.46–0.91)	0.19***	(0.1–0.37)	0.52***	(0.37–0.73)	0.46***	(0.33–0.65)
40+	0.01***	(0.00–0.11)	0.36*	(0.11–1.18)	0.24***	(0.14–0.41)	0.06***	(0.03–0.15)	0.19***	(0.1–0.35)	0.24***	(0.14–0.39)
No formal education	0.29	(0.06–1.42)	0.93	(0.49–1.75)	1.03	(0.8–1.32)	0.80	(0.4–1.61)	1.23	(0.86–1.75)	1.26	(0.88–1.8)
Primary education	1.34	(0.66–2.71)	0.58***	(0.41–0.84)	1.05	(0.88–1.27)	0.94	(0.68–1.3)	1.18	(0.92–1.53)	1.11	(0.9–1.37)
Secondary+ education (r)	1.00		1.00		1.00		0.00		1.00		1.00	
Poorest quintile	0.78	(0.27–2.29)	1.09	(0.66–1.82)	1.95***	(1.39–2.74)	1.40	(0.93–2.09)	1.30	(0.94–1.81)	1.70***	(1.2–2.41)
Poorer quintile	1.41	(0.56–3.55)	1.36	(0.82–2.27)	1.57***	(1.15–2.13)	1.49**	(1.01–2.2)	1.39**	(1.03–1.88)	1.89***	(1.32–2.72)
Middle quintile	0.67	(0.31–1.46)	1.14	(0.71–1.83)	1.32*	(0.98–1.77)	1.64***	(1.16–2.32)	1.25	(0.94–1.65)	1.13	(0.84–1.53)
Wealthier quintile	0.97	(0.45–2.1)	0.91	(0.54–1.56)	1.04	(0.79–1.37)	1.25	(0.9–1.72)	1.25	(0.94–1.66)	1.27	(0.94–1.7)
Wealthiest quintile (r)	1.00		1.00		1.00		1.00		1.00		1.00	
Intention to limit (r = space)	0.50**	(0.28–0.88)	1.31	(0.92–1.85)	0.91	(0.75–1.1)	0.94	(0.68–1.3)	0.70***	(0.58–0.85)	0.76***	(0.62–0.93)
Rural residence (r = urban)	1.01	(0.53–1.94)	0.99	(0.69–1.41)	0.80**	(0.67–0.96)	0.92	(0.71–1.2)	0.88	(0.74–1.06)	0.83	(0.65–1.05)
Never married (r = ever married)	0.90	(0.39–2.06)	0.89	(0.61–1.29)	0.77	(0.55–1.08)	0.71**	(0.53–0.94)	1.57***	(1.12–2.2)	0.72**	(0.56–0.94)

^*^Significant at p<0.1; ^**^p<0.05; ^***^p<0.01. (r) = Reference category.

NOTE: Hazard ratios are adjusted for all covariates in the model.

Women's socioeconomic status is associated with failure among pill, condom, withdrawal, and periodic abstinence users, with poorer women experiencing significantly higher hazards of failure than their wealthier counterparts (Table 2).^6^ Results are particularly strong for pill users, the poorest of whom have almost twice the hazard of failure of the wealthiest users. After adjusting for other variables in the model, there is no consistent relationship between failure and education. Strength of motivation to avoid pregnancy, as measured by intention to limit versus space, remains a significant correlate of failure for IUD, withdrawal, and periodic abstinence users. Urban versus rural residence appears to have little or no impact on contraceptive failure (except that rural pill users may have 20 percent lower hazards of failure than urban users), and patterns by marital status remain inconsistent.

#### Sensitivity Analyses

When limiting the sample to a 1‐year recall period (Appendix Table 2, N= 42,950 episodes of contraceptive use), we observed no statistically significant differences in failure rates as compared with our main analysis. There were too few cases of failure among the 1,109 implant episodes in this sample to produce failure rates, so we are unable to compare estimates for this method. In the second sensitivity analysis, keeping the 3‐year recall period but limiting the sample to surveys conducted in the last 10 years, the sample size was larger: 85,802 episodes of contraceptive use (Appendix Table 4). There were no statistically significant differences in overall failure rates between this and the main analysis. However, out of more than 100 comparisons between failure rates by method and characteristics, there were only two estimates that were significantly lower than our main analysis: those using withdrawal to limit, at 9.3 (95% CI 7.6–11.2) versus 13.0 (95% CI 11.4–14.8) in the main analysis, and ever‐married withdrawal users, at 13.7 (95% CI 12.4–15.3) versus 16.7 (95% CI 15.4–18.0) in the main analysis.

We compared hazard models between our main analysis and under the two conditions in the sensitivity analyses (i.e., restricted to a 1‐year recall period [Appendix Table 2], and restricted to surveys conducted in the past 10 years [Appendix Table 4]). Some statistically significant relationships in the main analysis were no longer significant in the restricted analyses (likely due to smaller sample sizes), and a small number of minor changes occurred (such as the relationship between pill use and intention to limit becoming significant in the sample of more recent surveys), but the findings between the main analysis and the sensitivity analyses were largely consistent.

### Limitations

Some covariates (particularly education and wealth quintile) were measured at the time of the survey, and not at the time of the episode of use. Since the episodes analyzed here took place in the most recent 3 years, it is unlikely that dramatic shifts occurred between categories in that time frame, but we acknowledge that there may be some misidentification in relationships with failure due to this limitation. The lack of major differences in the relationships with wealth and education in the 1‐year recall sensitivity analysis suggests that these measures are still applicable to events in the most recent 3 years.

These failure rate estimates are based on women's self‐reports, which are not validated in any clinical way, and may not precisely reflect women's actual contraceptive histories and contraceptive failures. Although we have tried to limit to surveys without evidence of underreporting of contraceptive use episodes, we are unable to adjust for potential underreporting of use episodes that end in abortion, as is done for estimates in the United States (Sundaram et al. 2017), so true failure rates could potentially be higher.

## DISCUSSION AND CONCLUSIONS

Consistent with other investigations of contraceptive failure, we found failure to be more common for users of short‐acting and user‐dependent methods, with more than 6 out of every 100 pill users and more than 8 out of 100 condom users becoming pregnant within the first year of use. Failure rates are particularly high for traditional methods, with more than 17 failures per 100 episodes of withdrawal and 19 failures per 100 episodes of periodic abstinence. These high levels of failure indicate an inability of family planning programs to help women and couples meet their reproductive intentions. High‐quality service provision including counseling that may decrease user errors, and widespread access to a wide variety of methods (including long‐acting methods that reduce opportunities for human error) are clearly warranted.

We find that contraceptive failure disproportionately affects the youngest and poorest women—in other words, women who may be the least able to care for an unintended child, obtain maternal health care, and access safe abortion services (Bankole et al. 2008; Gipson, Koenig, and Hindin 2008; Rasch and Kipingili 2009; Fung 2012; Sundaram et al. 2012; Joyce, Tan, and Zhang 2013). Given the anticipated increases in global numbers of contraceptive users (Brown et al. 2014), contraceptive failure is almost certain to become a more widespread phenomenon. The increasing contribution of contraceptive failures to the health and socioeconomic status of populations warrants a better understanding of this experience and how women resolve unintended pregnancy and the associated implications.

In this article, we have instituted a robust methodology to identify the highest‐quality nationally representative data available on contraceptive discontinuation. Our sensitivity analyses seem to confirm that the data used here produce estimates of contraceptive failure that are accurate and reliable. Using higher‐quality surveys appears to produce higher estimates of contraceptive failure rates than prior analyses of DHS calendar data that did not consider quality criteria (Ali, Cleland, and Shah 2012; Polis et al. 2016), though differences between estimates were not consistently statistically significant. The higher failure rates in the quality‐selected surveys suggest that failures and other discontinuations are likely to be underreported in the many surveys that collected less reliable calendar data. DHS calendar data quality should be considered in future studies using contraceptive calendar data.

One key finding from this analysis is the striking role that age plays in contraceptive failure, with the youngest users experiencing failure rates up to 10 times higher than older women for certain methods. These differences are dramatic enough that the discrepancies between our estimates and estimates from US data could potentially be explained by differences in the age composition of users. Newly published estimates of US failure rates based on National Survey of Family Growth (NSFG) data from 2006–10 are lower than previous US rates shown in Hatcher (2011) for every method, though results are still higher than results from this study. For example, the new condom failure rate estimate is 12.6 (SE 1.11) (Sundaram et al. 2017), which is still likely significantly higher than our estimate of 8.6. If the age distribution of contraceptive method users skews younger in the NSFG data on which the Hatcher (2011) and Sundaram and colleagues (2017) estimates are based, as compared with the sample analyzed here, the differences between estimates could potentially be explained entirely by compositional differences in the age structure of users. Though outside the scope of the present study, this is a clear area for further examination. In future research we plan to investigate age‐adjusted failure rates to facilitate comparisons across multiple populations with varying age structures.

Demographers have long used age‐specific data to model fertility, mortality, and other life experiences, but age patterns have not been widely incorporated in models of contraceptive failure, generally due to data limitations. Most modeling exercises that incorporate failure or use‐effectiveness use single values for entire methods or method categories, either based on US data (Kost et al. 2008; Hatcher 2011) or data from the Philippines in 1978 (Liang 1978; Bongaarts and Potter 1983; Bongaarts 2015) which are still used for estimating the Proximate Determinants model today (Bongaarts 2015). The age‐ and method‐specific failure estimates presented here provide a useful opportunity to refine existing models, particularly those that aim to understand or project fertility rates in low‐ and middle‐income countries. Such evidence could also be of potential use in clinical settings, to help women at different life stages better understand their own levels of risk. The findings presented here have direct applications for modeling approaches as well as for program and policy development worldwide.

Contraceptive use is among the clearest indicators of intention to avoid unintended pregnancy. Contraceptive use generally indicates that a woman or couple believes that their current situation—their age, marital status, recency of prior birth, number of living children, or financial situation—would make them unable to care for a child at that time. Users of contraceptive methods are attempting to intentionally plan the number and spacing of their children, and family planning programs must support them by helping potential users adopt methods that are most appropriate for them and by educating them about risks of contraceptive failure that are most relevant, including, when appropriate, the explanation and provision of age‐specific failure rates. As policies and programs encourage more women to adopt contraception, there must be an increasing focus on supporting women and couples in avoiding contraceptive failure, and providing support including safe abortion services when contraceptive failure does occur.

## Supporting information

Appendix Tables 1–4

## Footnotes

1 Appendix tables are available at the supporting information tab at wileyonlinelibrary.com/journal/sfp.

2 Although we were able to estimate failure rates for the contraceptive implant, failures are so rare that we were unable to assess correlates in multivariate models.

3 In some countries, fertility awareness methods (FAM) such as CycleBeads® are a part of the country's family planning program and such methods are included as separate categories in the DHS questionnaire. In these countries, the category of “periodic abstinence” excludes self‐identified FAM users. However, it is possible that some FAM users who self‐reported using “periodic abstinence” or “rhythm method” are included in this category.

4 Specifically, the percentage of contraceptive use episodes beginning during the period of observation that end in failure within the first 12 months. Although these are episode‐based, and not woman‐based, we find that on average only 10 percent of women report more than one episode of use of the same contraceptive method during the observation period. We therefore refer to the percentage of *women* rather than *episodes* for ease of interpretation.

5 When grouped into the poorest 60 percent (poorest 3 quintiles) and wealthiest 40 percent (wealthiest 2 quintiles), failure rates for pills, condoms, withdrawal, and periodic abstinence are significantly higher for women in the poorest 60 percent of the population than for women from wealthier households.

6 When grouped into the poorest 60 percent and wealthiest 40 percent, hazards of failure rates for pills, condoms, and periodic abstinence are significantly higher for women in the poorest 60 percent of the population than for women from wealthier households.

## ADDENDUM A EVALUATION OF CALENDAR DATA QUALITY

In evaluation of calendar data quality, we searched for ways to identify surveys that showed evidence of poor data quality. We examined the gap between the method‐specific contraceptive prevalence rate measured from retrospective calendar data and a previous current‐status estimate. Those estimates come from analyses described in Bradley, Winfrey, and Croft 2015. We excluded surveys that had outlying values on this measure for any of the contraceptive methods analyzed in this article.

In an attempt to quantify data quality for each survey, we looked for measures that could indicate that interviewers intentionally misrecorded information, usually to decrease their workload or the burden on their interviewees. One way to measure interviewer interference is to look for suspicious age patterns recorded for selected households. In most DHS surveys, interviews are conducted with all eligible women in the selected households—that is, women aged 15–49. If interviewers “push” 15‐year‐olds into age 14, or 49‐year‐olds into age 50, those women are no longer eligible and therefore do not need to be interviewed.

Absent some very unusual and specific birth or mortality patterns, we would expect to find roughly an equal number of 14‐year‐olds and 15‐year‐olds in a given population. In survey data, we would expect to find more people recorded as 15 years old than 14 years old, given uncertainty of birth dates and digit preferences for numbers ending in 0 or 5. We would not expect, however, to find a substantially higher number of 14‐year‐olds than 15‐year‐olds in a population, yet in some surveys the recorded age structure of the female population shows heaping on age 14. We detect this type of displacement by measuring:

- The ratio of women age 14:15 listed in the household schedule.
- The ratio of women age 50:49 listed in the household schedule.

We expect some normal heaping on age 50, given digit preference and uncertainty about dates of birth, particularly for older individuals. We wanted to classify surveys according to whether or not they had extreme values on each index, indicating potential problems with data quality. To detect extreme values on each index, we calculated the interquartile range (IQR) of the distribution of each index as p75 – p25. Following the standard statistical definition, we defined any value greater than p75 + 3/2^*^IQR to be an outlier.

Similar to the displacement of ages of women in eligible households, interviewers may displace dates of births of children, collected from interviewed women, outside the most recent 5‐year period, which typically coincides with the period covered by the calendar. Most DHS surveys ask a lengthy set of questions about the antenatal care, delivery assistance, vaccination record, and so on, about every child born in the past 5 calendar years prior to the survey. Children who were born 6 or more years before the survey, however, are not eligible for this set of questions (asked to the mother). Interviewers may displace births that occurred in the past 5 years to the prior year, thereby artificially shortening the questionnaire (see Bradley 2015). We detect this type of displacement by measuring:

- The ratio of the number of births in the calendar year six years prior to survey: the number of births in the calendar year five years prior to survey.

To assess additional reporting issues in the calendar portion of the survey, we investigated heaping with several measures. We believe that, in most cases, there is not a reason to expect contraceptive use episodes to be exactly 12 months in length, rather than 11 or 13 months. Rather, if a woman reports that she used a method for “a year” and the interviewer does not probe for additional detail, the duration of use would be recorded as 12 months. This is particularly problematic if the episode was actually shorter than a full year, because this could bias 12‐month failure rates. Graphical analysis suggested that reported durations of contraceptive use were strongly heaped on 12 or 6 months in some surveys. We therefore measured:

- The ratio of contraceptive use episodes reported to be 12 months duration: the average of episodes reported to be 10, 11, 13, or 14 months duration.
- The ratio of contraceptive use episodes reported to be 6 months duration: the average of contraceptive use episodes reported to be 4, 5, 7, or 8 months duration.

The heaping ratios were all calculated separately for each contraceptive method. Because the injectable is typically effective for three months, we expect reported durations of injectable use to be heaped on 3‐month intervals (e.g., 3 months, 6 months, 9 months, 12 months, and so on). The 12‐month duration heaping index for injectables was therefore calculated as the ratio of episodes of 12‐month duration: the average of episodes of 9 or 15 months duration, and the 6‐month heaping index for injectables was calculated as the ratio of episodes of 6 months duration: the average of episodes of 3 or 9 months duration.

Graphical analysis also suggested that the start date of contraceptive use was strongly heaped on the month of January in many surveys. While this could in fact represent increases in availability of certain contraceptive methods, if stocks were resupplied at the beginning of the calendar year, we see no reason to believe that substantially more withdrawal or condom users began use in January rather than in December or March. It seems more plausible that women reported they began a use episode “in 2010,” and the interviewer recorded the episode to have begun in January of that year. To indicate surveys in which the start date of contraceptive use episodes were strongly heaped on the month of January we measured:

- The ratio of contraceptive use episodes reported to have begun in January: the average of contraceptive use episodes reported to have begun in February or March.^7^

Each index was calculated for each survey and, where applicable, each contraceptive method. To select the most reliable data available, we eliminated from our sample any survey that had outlying values on any of these indices or measures.

## ADDENDUM B LIFE TABLE AND CONFIDENCE INTERVAL CALCULATIONS

Life table failure rates are equivalent to cumulative probabilities. We calculate the probability of failing in each month *x*, conditional on not having failed in the previous month. The probability of “surviving” (i.e., not experiencing contraceptive failure in month *x*), is the complement of the failure probability. A failure rate is equivalent to 1—the cumulative product of the monthly conditional survival probabilities.

Specifically, we construct the cumulative probability of failure by month 12, or one‐year failure rate, as the complement of

(1) $$S_{12}=\prod_{x=1}^{12}\left(1-p_{x}\right)$$

where *S_12_* is the cumulative probability of “surviving” (i.e., not experiencing contraceptive failure) to and through month 12 of contraceptive use, and *p_x_* is the conditional probability of failure in month *x* in 1, 2, … 12, given that the user did not fail in any prior month.

We calculate the monthly conditional probabilities using logit regression. A logit regression of failure on dummy variables for each month *x* gives the inverse logit of the conditional probability of failure in each month. Substituting in logistic regression estimates $invlogit({\hat{B}}_{x})$ and taking logs gives

(2) $$\log\;\left({\hat{S}}_{12}\right)=\sum_{x=1}^{12}\log\;\left(1+e^{\hat{\hat{B}}x}\right)$$

The estimates of ${\hat{B}}_{x}$ are produced using Stata's svy: logit commands, giving

(3) $$\hat{B}∼N\left(B,\varepsilon \right)$$

An advantage to this regression‐based approach for failure rate calculation is that estimates of variance can be calculated taking full account of the sample design, as noted by Abatih and colleagues (2008) including the stratified, clustered sample design used in DHS surveys. We implement this by using Stata's svy suite of commands to produce the failure probabilities and associated variance‐covariance matrix. The variance and confidence intervals around the cumulative failure probability are then estimated using the Delta Method (Oehlert 1992; Fishman 2015). We define

(4) $$g\left(\hat{B}\right)=\sum_{x=1}^{12}\log\;\left(1+e^{\hat{\hat{B}}x}\right)$$

According to the Delta method (Oehlert 1992),

$$g\left(\hat{B}\right)∼\;N\left(g\left(B\right),V\right)$$

Where $V=A^{\prime }*\varepsilon *A$

(5) And A=∂gB∂B1∂gB∂B2⋮∂gB∂B12

We need to ensure that the lower and upper bounds of the confidence interval around 1‐S_12_ are constrained to lie in (0,1). We do this by using a log(‐log) transformation, noting that the variance of $\log(-\log({\hat{S}}_{12}))$ is approximated with the Delta Method as

(6) $$\theta =\;\frac{1}{{\left[-\log\left({\hat{S}}_{12}\right)\right]}^{2}}*V$$

The confidence interval for $\log(-\log({\hat{S}}_{12}))$ is then

(7) $$\log\left(-\log\left({\hat{S}}_{12}\right)\right)\pm z_{0.975}*\sqrt{V}$$

And the CI for 1‐ ${\hat{S}}_{12}$ is estimated as

(8) $$1-\exp\left(-\;\exp\left[\log\left(-\log\left({\hat{S}}_{12}\right)\right)\pm z_{0.975}*\sqrt{V}\right]\right)$$
